# Proteomic Sequencing of Stellate Ganglions in Rabbits With Myocardial Infarction

**DOI:** 10.3389/fphys.2021.687424

**Published:** 2021-12-16

**Authors:** Lijun Cheng, Xinghua Wang, Hongda Chou, Tong Liu, Huaying Fu, Guangping Li

**Affiliations:** Tianjin Key Laboratory of Ionic-Molecular Function of Cardiovascular Disease, Department of Cardiology, Tianjin Institute of Cardiology, The Second Hospital of Tianjin Medical University, Tianjin, China

**Keywords:** stellate ganglion, myocardial infarction, TMT quantitative proteomic sequencing, rabbit, KEGG analysis, GO analysis

## Abstract

The stellate ganglion (SG) of the autonomic nervous system plays important role in cardiovascular diseases (CDs). Myocardial infarction (MI) is associated with sustained increasing cardiac sympathetic nerve activity. Expressions and functions of proteins in SG tissue after MI are remaining unclear. This study is to explore the expression characteristics of proteins in SGs associated with MI. Japanese big-ear white rabbits (*n* = 22) were randomly assigned to the control group and MI group. The MI model was established by left anterior descending coronary artery ligation and confirmed by serum myocardial enzymes increasing 2,3,5-triphenyltetrazolium (TTC) staining and echocardiography. The expressions of proteins in rabbit SGs after MI were detected using tandem mass tags (TMT) quantitative proteomic sequencing. There were 3,043 credible proteins were predicted in rabbit SG tissues and 383 differentially expressed proteins (DEPs) including 143 upregulated and 240 downregulated proteins. Gene ontology (GO) and Kyoto Encyclopedia of Genes and Genomes (KEGG) analysis showed that the DEPs involved in adrenergic signaling in cardiomyocytes, positive regulation of ERK1 and ERK2 cascade, and other biological processes. Three kinds of proteins directly correlated to CDs were selected to be validated by the subsequent western blot experiment. This study first identified the characterization of proteins in rabbit SG after MI, which laid a solid foundation for revealing the mechanism of roles of SG on the MI process.

## Introduction

Myocardial infarction (MI) is a common cardiovascular disease (CD), which seriously endangers the health of middle-aged and elderly people. The stellate ganglion (SG) in the autonomic nervous system provides sympathetic outflow and plays an integrative role in regulating cardiovascular function (Yu et al., 2017a). The SG has been implicated in the pathogenesis of various CDs. Following MI, SG was involved in the process of catecholamine released and promoted cardiac arrhythmogenesis (Richardt et al., 2006; Wu et al., 2012). Also, morphological, neurochemical, and electrophysiological changes in the SG neurons were observed in the areas distant from the infarct zone (Ajijola et al., 2015; Cheng et al., 2016), which may be important factors leading to ventricular arrhythmia after MI. The results of clinical and animal experiments showed that SG blockade can improve and treat CDs (Gu et al., 2012; Meng et al., 2017; Yu et al., 2017b), but it had adverse complications (including Horner's syndrome, hyperhidrosis, and paraesthesia) (Goel et al., 2019). Many proteins of SG participated in the regulation of cardiac function. The targeted intervention of these proteins can improve cardiac function after myocardial ischemia or infarction. For example, previous studies have shown that the characteristics of ion channels in sympathetic ganglion neurons changed significantly after myocardial ischemia and infarction (Cheng et al., 2016, 2018). Blocking the ion channel of SG neurons can significantly attenuate ischemia-induced ventricular arrhythmia by suppressing the SG activity (Yu et al., 2017a). In addition, the neural chemorepellent Semaphorin 3a overexpression in the SG ameliorated the inducibility of ventricular arrhythmias after MI through attenuation of neural remodeling within the cardiac neuraxis (Yang et al., 2016). P2X7 inhibition can prevent the pathophysiologic processes mediated by P2X7 receptors in the SG after myocardial ischemic injury (Zou et al., 2016). So, it is necessary to find the key proteins in SGs regulating cardiac function, to reduce sympathetic overactivation after MI by target intervention of these proteins safely and effectively. However, analyzing the proteins in ganglia after MI comprehensively and systematically is absent. Discovering differentially expressed proteins (DEPs) in SG after MI is an urgent problem to be solved.

To find DEPs in SGs after MI, in this study, we established the rabbit MI model to reveal the expression characterization of proteins in MI rabbit SG tissues by tandem mass tags (TMT) quantitative proteomic sequencing and screening out the key proteins that are regulating the process of MI in SG tissues, which will provide a reliable experimental basis for finding effective intervention targets in SGs after MI.

## Materials and Methods

### Experimental Animals and Protocol

All animal studies were approved by the Animal Ethical and Welfare Committee of the Chinese Academy Medical Sciences Institute of Radiation Medicine. Japanese big-ear white male rabbits weighing 400–600 g were obtained from Tianjin Yuda Experimental Animal Co. Ltd. (Tianjin, China). They were randomly divided into the control group and MI group (each group *n* = 11). MI injury was induced by ligating the left anterior descending coronary artery (LADCA). First, rabbits were anesthetized using 3% pentobarbital sodium (1 ml/kg) intravenously via the marginal ear vein. A left thoracotomy was performed. Then, the LAD was identified and it was ligated and the thorax was closed. The rabbits in the control group underwent an identical surgical procedure without ligation. Four rabbits died during the experimental process. Randomly selected rabbits were replenished for the experiment, and the number of animals in each group was 11. After 24 h of surgery in two groups, blood samples were obtained. After 7 d of surgery, the animals were sacrificed, hearts and SG tissues were obtained for the following experiments. MI was confirmed using 2,3,5-triphenyltetrazolium chloride (TTC) staining, increasing the levels of the three serum myocardial enzymes, and echocardiography.

### Echocardiography

After MI surgery, rabbits in the two groups were anesthetized using 3% pentobarbital sodium to measure transthoracic echocardiography. Echocardiography parameters, including left atrial diameter (LAD), left ventricular end-diastolic dimension (LVDD), left ventricular end-systolic dimension (LVSD), and left ventricular ejection fraction (LVEF), were obtained using a specified small animal ultrasound system (VisualSonics Vevo 2100, USA).

### Analyzing Myocardial Enzymes

Blood samples (1-2 ml) were collected from the jugular veins of rabbits in the control and MI groups. Sera were separated using centrifugation and stored in the refrigerator at −20°C. Serum myocardial enzymes including creatine kinase isoenzyme (CK-MB), creatine kinase (CK), and lactate dehydrogenase (LDH) were analyzed following the instructions of the manufacturer (Nanjing Jiancheng Bioengineering Institute, China).

### TTC Staining

TTC staining was performed to assess the myocardial infarct size. In brief, the animal was fixed on the table, and the heart was taken out quickly. After washing the residual blood from the heart with the phosphate buffer, the heart was frozen in the refrigerator at −20°C for 30 min. Then the heart was transected into 2–3 mm thick sections. The sections were incubated in 0.5% TTC solution (30 min, 37°C) and fixed with 10% formalin. Normal myocardium was stained in red color, and the infarcted myocardium was stained in white color. Then the stained myocardium was photographed, and the infarct size was calculated.

### TMT Quantitative Proteomic Sequencing and Bioinformatics Analysis

In this study, we used the “mix the samples” method to reduce the variation in a single group. In contrast, the SG tissue of a single rabbit is small and cannot meet the weight requirements for sequencing analysis; the SG tissues of two rabbits need to be mixed. Therefore, SGs of two rabbits in the control group were randomly mixed; six rabbits were divided into con1, con2, and con3 (*n* = 3). It was the same as in the MI group; six rabbits were divided into MI1, MI2, and MI3 (*n* = 3). TMT quantitative proteomic sequencing and subsequent bioinformatics analysis were performed using the Shanghai Luming Biological Technology Co. Ltd. (Shanghai, China). Briefly, the process of TMT quantitative proteomic sequencing was as follows: Frozen samples were lysed with 300 μl lysis buffer supplemented with 1 mM PMSF (Amresco, USA). After sonication, the samples were centrifuged to remove insoluble particles and precipitation. Protein concentration was determined using the BCA Protein Assay Kit (ThermoScientific, USA). The amount of protein can be analyzed using the SDS-PAGE electrophoresis method. The 100 μg proteins were hydrolyzed into peptides using 2 μl sequencing-grade trypsin (1 μg/μl). Digested peptides were labeled using a 41 μl TMT reagent (ThermoFisher, USA) and incubated using 8 μl 5% hydroxylamine (Sigma, USA) to terminate the reaction. The High Performance Liquid Chromatography (RPLC) analysis was performed on an 1100 HPLC System (Agilent, USA) using an Agilent Zorbax Extend RP column (5 μm, 150 mm × 2.1 mm). Mass spectrometry analysis was performed using a Q Exactive Mass Spectrometer (Thermo, USA). Samples were loaded and separated using Acclaim RepMap 100 column [100 μm × 2 cm, RP-C18, Acclaim RepMap (Thermo Fisher, USA)] and then separated using Acclaim RepMap RSLC column (15 cm × 75 μm, RP-C18, Thermo Fisher) on an EASY-nLC 1200 system (Thermo, USA).

### Western Blot

SG tissues were ground to extract total protein. The total protein concentration was determined using the BCA Protein Assay Kit (Cwbio, China). Equal amounts of proteins were subjected to 10% SDS-PAGE and transferred onto polyvinylidene fluoride (PVDF) membranes (Millipore, Billerica, MA, USA). Membranes were blocked using 5% skimmed milk and subsequently incubated overnight at 4°C with anti-calmodulin (CALM) (Bioss Antibodies, China; 1:1,000), anti-tropomyosin 1 (TPM1) (Boster, China; 1:1,000), anti-tropomyosin 2 (TPM2) (Bioss Antibodies, China; 1:1,000), and anti-β-actin (TransGen Biotech, China; 1:5,000). Then, the membranes were incubated using secondary antibodies, including goat anti-Mouse IgG (H+L) HRP (Promega Corporation, USA; 1:5,000), or goat anti-rabbit IgG (H+L) HRP (Promega Corporation, USA; 1:5,000). Last, protein signals were assessed and analyzed using the ECL western blot detection system (Millipore, Billerica, MA, USA).

### Statistical Analysis

Proteome Discoverer (version 2.2) was used to search all of the Q Exactive raw data thoroughly against the sample protein database. The database used was uniprot-proteome_UP000001811-Oryctolagus cuniculus (Rabbit) (Strain Thorbecke inbred) database. The search settings were selected as follows: the sample type was TMT 6 plex (Peptide Labeled), the cysteine alkylation was performed using iodoacetamide, digestion was done using trypsin, and the instrument used was Q Exactive. A global false discovery rate (FDR) was set to 0.01.

According to the search results of the protein database, the original data were obtained. The credible proteins and DEPs were selected, and the subsequent biological information function analysis was carried out based on the DEPs. Several common databases were used for functional annotation analysis of credible proteins. Gene ontology (GO) analysis, Kyoto Encyclopedia of Genes and Genomes (KEGG) analysis, and protein interaction analysis were done in the DEPs. Correlation analysis, expression pattern clustering map, and heat map were performed in the control and MI group proteins.

Data were analyzed using Proteome Discoverer (Thermo Scientific, USA), Origin 6.0 (OriginLab Inc., USA), SPSS 17.0 (SPSS, USA). All data were treated with a normal distribution test and variance homogeneity test. The variables were expressed as mean ± standard deviation (SD) and statistically analyzed using a *t*-test. *P* < 0.05 were considered statistically significant.

## Results

### Validation of the MI Model

**Figure 1:**
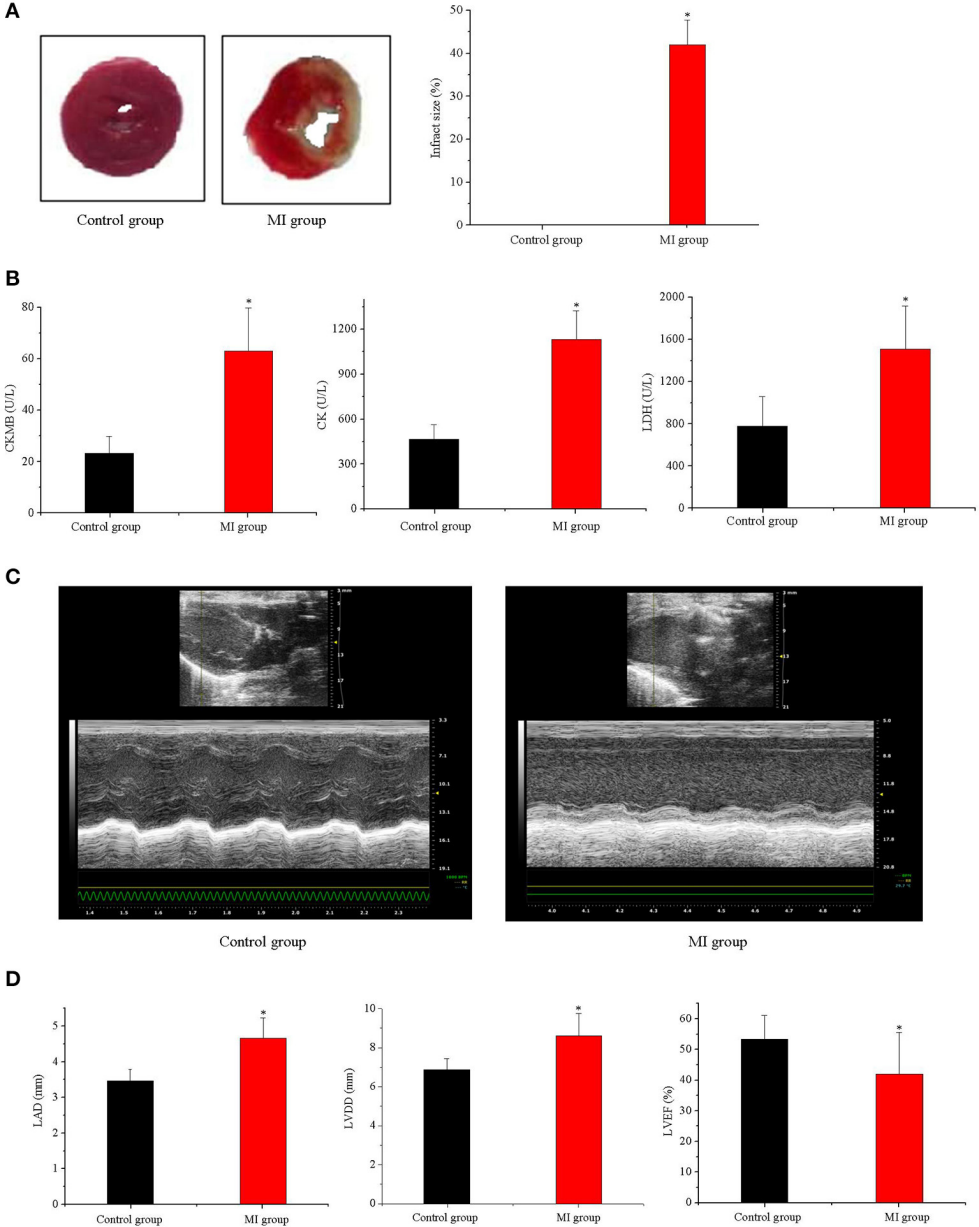
Validation of the MI model. **(A)** TTC staining of MI and the infract size (*n* = 6, *p* < 0.05); **(B)** CK-MB, CK, and LDH in serum in control and MI group (*n* = 11, *p* < 0.05); **(C,D)** the cardiac function in the MI group decreased (*n* = 11, *p* < 0.05). Each point represents mean ± SD, *n* is the number of animals, ^*^p < 0.05 compared with the control group.

The MI model was confirmed and assessed using TTC staining, higher serum myocardial enzymes, and a decrease in cardiac function. According to the experimental results of TTC staining, there was no MI area in the control group; in contrast, the mean infarct area in the MI group was (42 ± 5.7)% (*n* = 6, *p* < 0.05; Figure 1A). The levels of serum myocardial enzymes (CK-MB, CK, and LDH) in the MI group were increased significantly compared with those in the control group (*n* = 11, *p* < 0.05; Figure 1B). Compared with the control group, the cardiac chamber dilation and left ventricle function decreased in the MI group, which manifested in the increasing of the inner diameter of the atrium and ventricle and the decreasing of left ventricle ejection fraction (*n* = 11, *p* < 0.05; Figures 1C,D).

### General Characteristics of Proteins Obtained Using Sequencing

**Figure 2:**
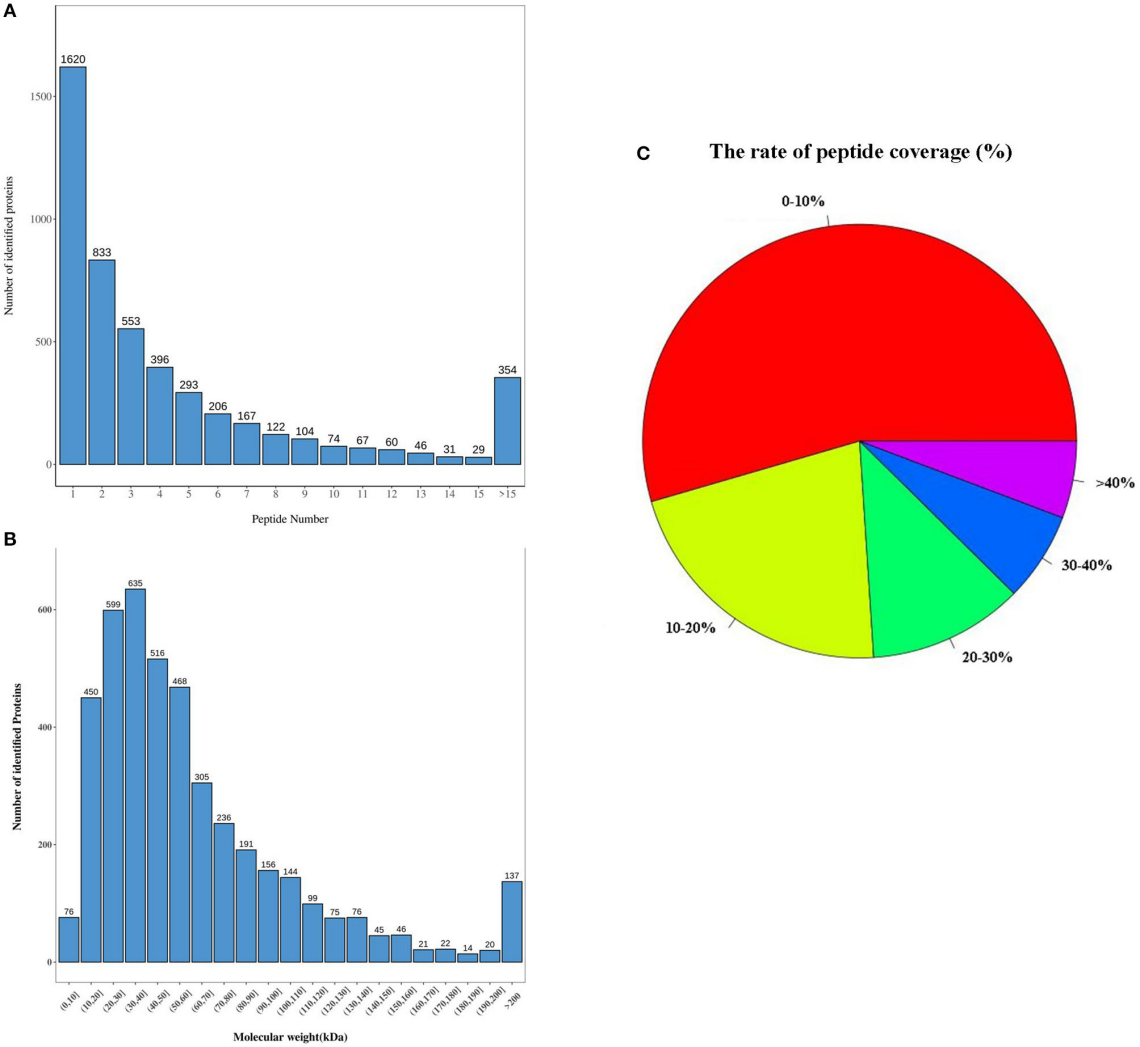
General characteristics of proteins obtained by sequencing. **(A)** The distribution of peptide number corresponding to each qualitative protein; **(B)** the number of proteins corresponding to different molecular weights; **(C)** the rate of peptide coverage (%). Red, the proportion of protein with peptide coverage of 0–10%; yellow, the proportion of protein with peptide coverage of 10–20%; green, the proportion of protein with peptide coverage of 20–30%; blue, the proportion of protein with peptide coverage of 30–40%; purple, the proportion of protein with peptide coverage of >40%. There were three samples in each group of the control and MI groups (*n* = 3).

The distribution of peptide numbers corresponding to each qualitative protein in the original off-line data is shown in Figure 2A. The protein numbers corresponding to different molecular weights are displayed in Figure 2B. In the qualitative process, we compared each peptide segment with that in the background database and obtained the coverage index of the peptide relative to the complete protein sequence using the database search software and made statistics according to the coverage index, as shown in Figure 2C.

### Differentially Expressed Proteins

**Figure 3:**
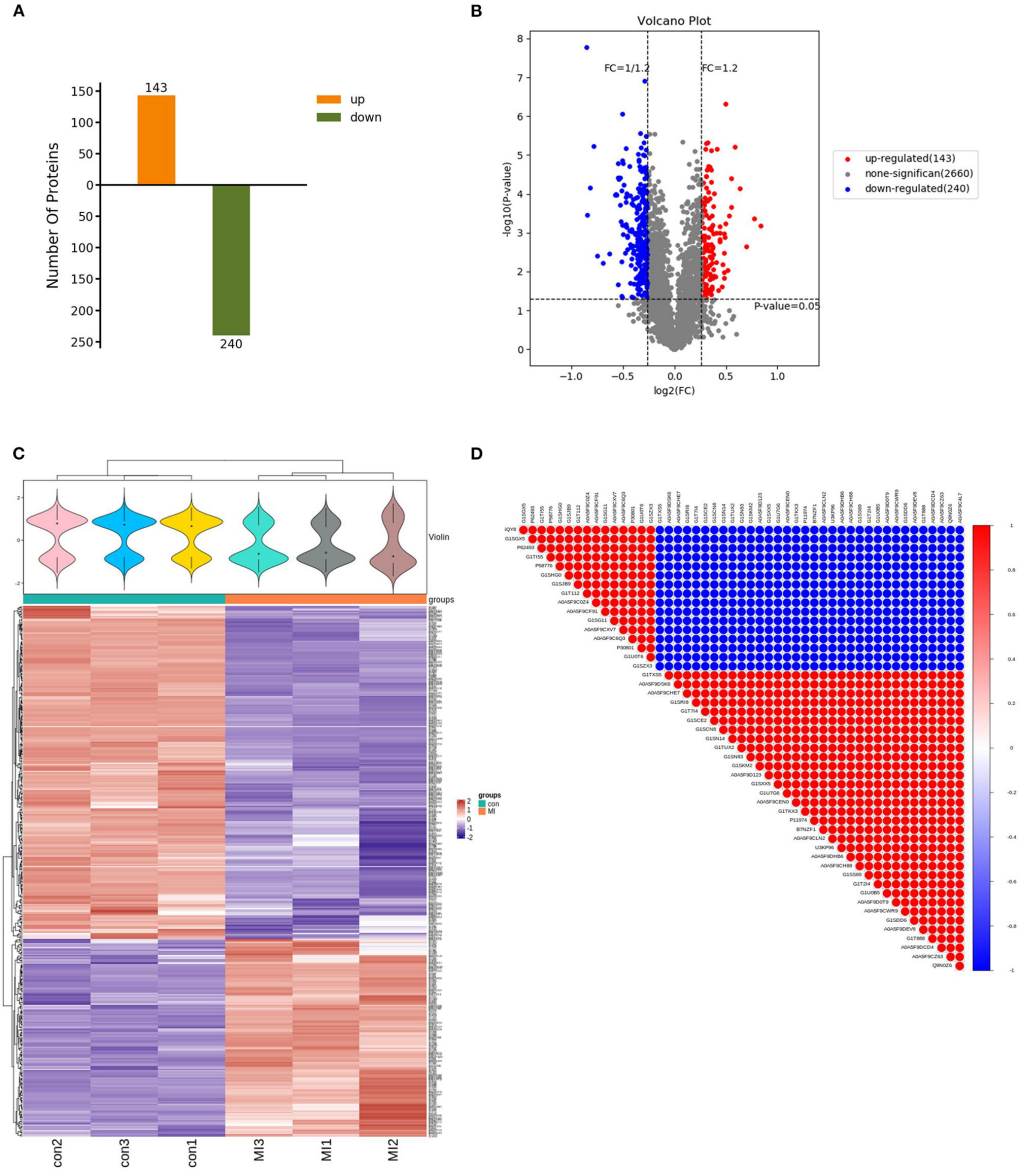
Characteristics of DEPs. **(A)** Totally 383 DEPs in the MI group, including 143 upregulated and 240 downregulated proteins (the abscissa is the comparison group, and the ordinate is the number of differential proteins); **(B)** volcano map filtering out the DEPs [the abscissa of the volcano map is log_2_(FC)]. The farther its value is from point 0, the greater the difference is. It is upregulated on the right and downregulated on the left. The ordinate is –log_10_(*p*-value). The farther the ordinate value from point 0, the greater is the difference. The blue dots indicate downregulated DEPs, red dots indicate upregulated DEPs, and black dots indicate non-significant DEPs; **(C)** cluster heatmap of the 383 differentially expressed proteins (the violin graph in the upper part is a combination of a box plot and density graph). The flatter the violin box, the more concentrated the data. The outline of the box reflected the probability distribution of the expression value. Different color filled in represents different samples. The “+” in the middle of the violin graph indicates the median of the data; the vertical axis is the protein expression level. Below the violin is the column annotations of the heat map, the samples in the same group correspond to the same color block annotation. The clustering heat map below is clustered according to protein expression level. The red color indicates high expression protein, the blue color indicates low expression protein, and each row indicates the expression level of each protein in each different group, and each column represented the expression of all differential proteins in each group. **(D)** The correlation analysis diagram of the top 50 DEPs (red is a positive correlation, blue is a negative correlation. The darker color means the greater correlation). There were three samples in each group of control and MI group (*n* = 3).

After using the database to retrieve the original data, the search results were screened for credible proteins according to the unique peptide ≥1, and the proteins with expression values of more than 50% in the samples were retained. The mean of the samples in one group was filled in the proteins with missing values <50%. Credible proteins were obtained through median normalization and log2 logarithmic conversion. There were 3,043 credible proteins expressed in the SG tissues of rabbits. Based on credible protein, two standards were selected to calculate the difference between samples. Fold change (FC) was used to evaluate the change in the expression level of a protein between samples. The *p*-value was calculated using a *t*-test. The DEPs between the control and MI groups were screened using fold change ≥1.2 and *p* < 0.05. Compared with the control group, there were 383 kinds of DEPs in MI rabbits, which included 143 upregulated and 240 downregulated proteins (shown in Figure 3A). By using the Volcano map, we filtered out the DEPs as shown in Figure 3B.

Unsupervised hierarchical clustering was based on the R language. The cluster heatmap of the control and MI groups is shown in Figure 3C. By using the Pearson algorithm, we analyzed the correlation between the DEPs. The correlation analysis diagram of the top 50 DEPs is shown in Figure 3D. The closer the correlation coefficient to 1, the higher the similarity of expression patterns between proteins.

### GO and KEGG Analysis of the Differentially Expressed Proteins

After obtaining the DEPs, the DEPs were enriched and analyzed using GO/KEGG to describe their functions. In GO/KEGG functional enrichment analysis method, all credible proteins were considered for the background list and the differential protein list was considered as the candidate list screened from the background list. The hypergeometric distribution test was used to calculate the *p*-value, and the *p*-value was corrected using Benjamin-Hochberg multiple tests to obtain FDR.

**Figure 4:**
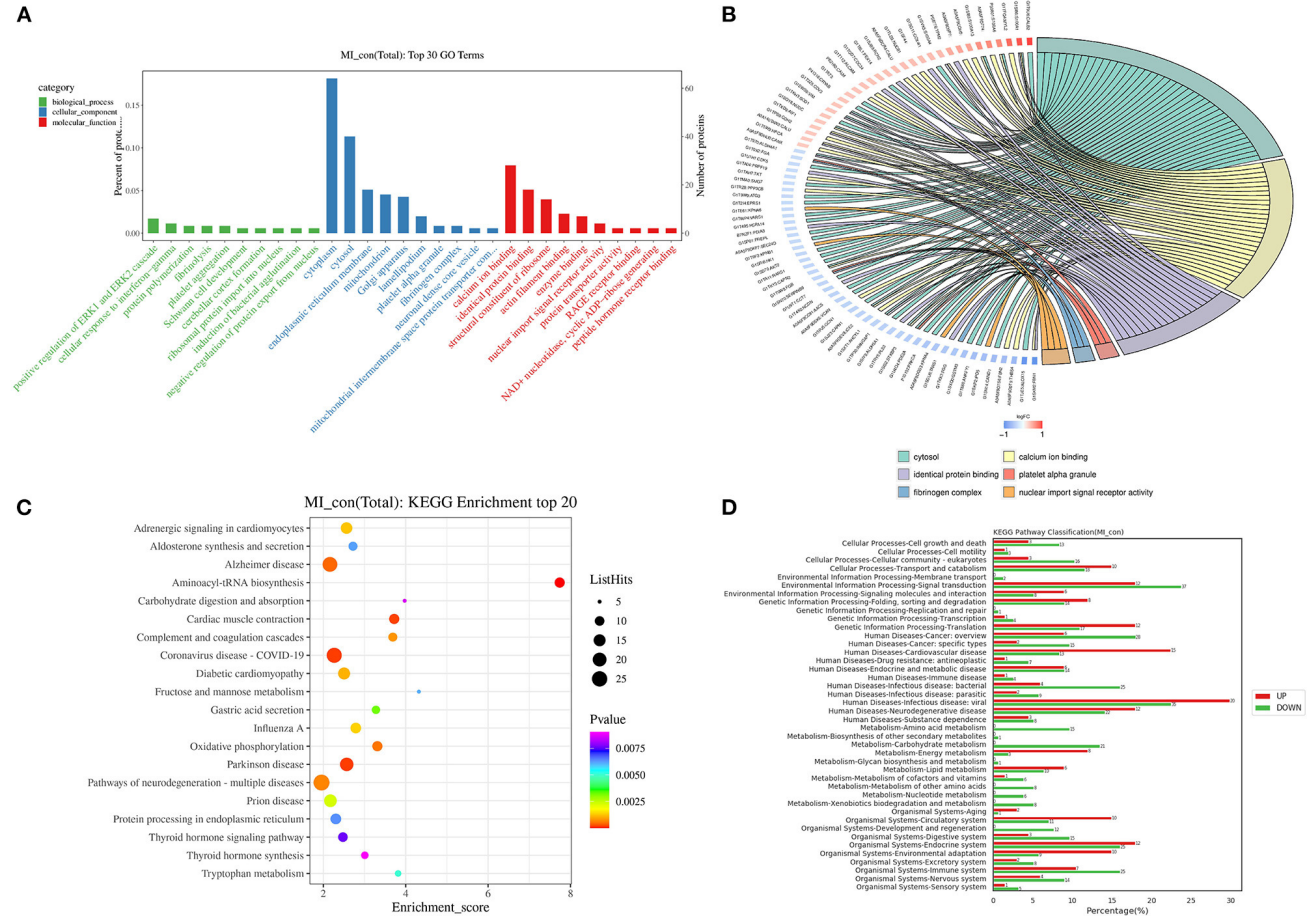
Go and KEGG analysis of the DEPs. **(A)** GO annotation analysis of proteins with top 10 in “biological process,” “cellular component,” and “molecular function” (the *x*-axis is the name of GO entry, the *y*-axis is the protein quantity and its percentage of the corresponding item); **(B)** go enrichment analysis chord diagram (the protein is on the left: gene name, the red color indicated upregulation, the blue color indicated downregulation. The selected GO term is on the right); **(C)** KEGG enrichment of top 20 (the *x*-axis is the enrichment score, and the *y*-axis is the pathway information of top 20. The larger the bubble means more differential protein numbers. The color of bubbles changed from purple-blue-green to red. The smaller the *p-*value, the greater the significance); **(D)** the distribution of upregulated and downregulated proteins at KEGG level 2 [the *x*-axis is the number and ratio (%) of differentially upregulated and downregulated proteins annotated to each level 2 metabolic pathway. The *y*-axis is the name of the level 2 pathway, and the number on the right side of the column represents the number of DEPs annotated to the level 2 pathway].

To gain a deeper understanding of these DEPs after MI, the GO analysis was performed to analyze the function of these DEPs. The proteins were categorized based on the characters of “biological process,” “cellular component,” and “molecular function.” The first 3 categories of the biological process were “positive regulation of ERK1 and ERK2 cascade,” “cellular response to interferon-gamma,” and “protein polymerization,” respectively. The first 3 categories of the cellular component were “cytoplasm,” “cytosol,” and “endoplasmic reticulum membrane,” respectively. The first 3 top categories of molecular function were “calcium ion binding,” “identical protein binding,” and “structural constituent of the ribosome” (Figure 4A). The first 6 items with the number of DEPs excesses than 3 and <50 in each comparison group were selected, which are sorted from large to small according to the –log_10_ *p*-value corresponding to each item, and the GO enrichment analysis chord representing the relationship between the selected GO term and the corresponding differential protein list is shown in Figure 4B. The KEGG analysis of these DEPs was also carried out to systematically analyze the regulatory role of these proteins. Top 20 KEGG enrichment proteins are shown in Figure 4C, and their roles include “adrenergic signaling in cardiomyocytes,” “aldosterone synthesis and secretion,” “Alzheimer's disease,” “aminoacyl-tRNA biosynthesis,” and other processes. The distributions of differentially upregulated and downregulated proteins at KEGG Level 2 are shown in Figure 4D.

### Verification of the Proteins by Western Blot

**Figure 5:**
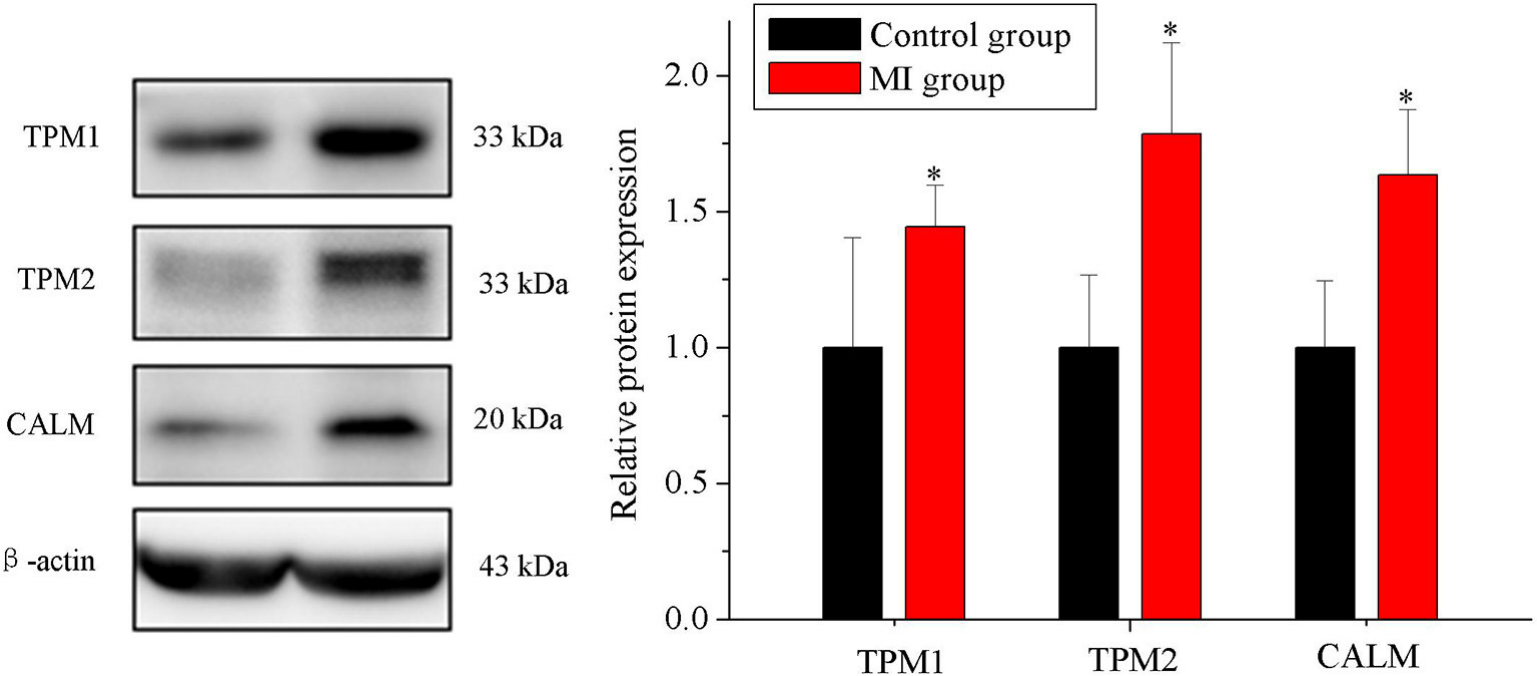
Verification of the proteins using Western blot. Expression of TPM1, TPM2, and CALM in the control and MI groups (*n* = 5, *p* < 0.05). Each point represents mean ± SD; *n* is the number of animals; ^*^p < 0.05 compared with the control group.

Among the DEPs, we focused on the DEPs related to human CDs. We screened 7 items in KEGG results by classification_level1 (“human disease”) and classification_level2 (“CDs”). Proteins in these items were involved in the occurrence and development of human CDs. We presented DEPs related to CDs of human diseases. Three of the proteins (meeting the following conditions: at least involved in two items, fold change >1.2 or fold change <0.8, and score sequence HT of protein >50) were selected for verification using Western blot experiment, including tropomyosin 1 (TPM1), tropomyosin 2 (TPM2), and calmodulin (CALM) in Table 1. Western blot results showed that three proteins expressions were significantly increased in MI group rabbits, which is consistent with the results of TMT quantitative proteomic sequencing analysis (*n* = 5, *p* < 0.05; Figure 5).

**Table 1:** KEGG analysis related to CD of human diseases.

**Id**	**Classification_level1**	**Classification_level2**	**Term**	**Protein**	***p*-value**
ocu05415	Human Diseases	CD	Diabetic cardiomyopathy	G1T359: NDUFS1, A0A5F9C1W2: ATP2A2, G1U7G6: G1TVE5: CAMK2D, G1SDD6: AGT, P05772: PRKCB, P10102: PRKCA, G1SD70: AKT2, G1SQA8: ATP5F1B, G1SG11: COX4I1, G1SXI9: O79431: MT-ATP8, G1TMH7: G1TPV7: G1SN66: NDUFB6	0.00106
ocu05418	Human Diseases	CD	Fluid shear stress and atherosclerosis	G1U9R0: G1SXQ0: GSTM3, G1SXP3: G1SD70: AKT2, P62160: CALM	0.51084
ocu05410	Human Diseases	CD	Hypertrophic cardiomyopathy	A0A5F9C1W2: ATP2A2, G1SDD6: AGT, P58776: TPM2, A0A5F9CLU9: TPM1, G1TW48: MYH7, G1TFQ4: MYL2	0.12617
ocu05412	Human Diseases	CD	Arrhythmogenic right ventricular cardiomyopathy	A0A5F9C1W2: ATP2A2, G1TPS9: CDH2	0.80004
ocu05414	Human Diseases	CD	Dilated cardiomyopathy	A0A5F9C1W2: ATP2A2, G1SDD6: AGT, P58776: TPM2, A0A5F9CLU9: TPM1, G1TW48: MYH7, G1TFQ4: MYL2	0.37173
ocu05416	Human Diseases	CD	Viral myocarditis	P41110: EIF4G1, G1TW48: MYH7, G1U0B4: A0A5F9CVI4	0.61311
ocu05417	Human Diseases	CD	Lipid and atherosclerosis	G1TVE5: CAMK2D, P10102: PRKCA, G1TRZ8: PPP3CB, G1SD70: AKT2, P62160: CALM, G1U0B4	0.57190

### Proteins Interaction Network

**Figure 6:**
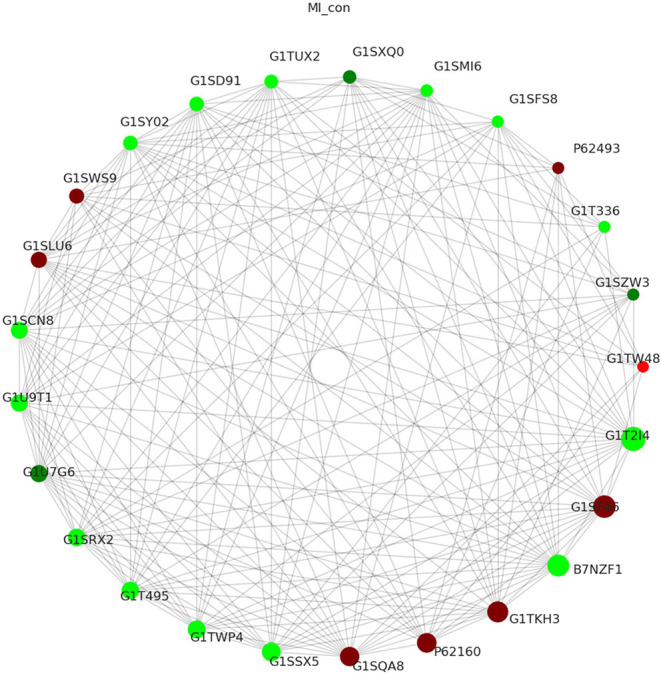
Proteins interaction network shows the interaction between DEPs after MI (the circles in the figure represented differential proteins, the red color represented upregulated protein, and the green color represented downregulated protein. The size of the circle represented the degree of connected correlation, and the larger the circle, the higher the connected correlation).

STRING database is a database for predicting functional correlation between proteins. In the STRING database, we analyzed the interaction relationship of DEPs following MI, visualized the first 25 nodes in terms of node connectivity using the python “networkx” package, and displayed them with protein IDs and gene names (Figure 6). Proteins interaction network analysis demonstrated that one protein interacted with another or more other proteins directly or interacted with many proteins indirectly. These results were used to construct a complex and extensive protein regulatory network.

## Discussion

We presented the profiled proteins expression in rabbit SG tissues using the TMT quantitative proteomic sequencing in this study first. We identified the DEPs in MI rabbit SG tissues and analyzed the DEPs using GO, KEGG, and proteins interaction analysis. Three of the proteins (meeting the following conditions: at least involved in two items, fold change >1.2 or fold change <0.8, and score sequence HT of protein >50) were selected for verification using Western blot experiment, including TPM1, TPM2, and CALM.

The MI induced a series of changes including autonomic nervous system remodeling in the heart (Richardson et al., 2015). The SG in the autonomic nervous system played an integrative role in regulating cardiovascular function (Yu et al., 2017b; Boukens et al., 2020). Previous studies have shown that MI can lead to neural remodeling in the SG, including remodeling in the morphology, neurochemistry, electrophysiological remodeling, and sympathetic hyperinnervation (Zhou et al., 2008; Ajijola et al., 2015; Wang et al., 2015; Nakamura et al., 2016), which contributed to MI-induced ventricular arrhythmias (Han et al., 2012; Sheng et al., 2018). Interventions to reduce SG activity can improve cardiac function and reduce ventricular arrhythmogenicity (Gu et al., 2012; Meng et al., 2017; Xiong et al., 2018; Zhou et al., 2019). However, the roles and mechanisms of SG in MI have not been clarified.

Previous clinical and basic studies have shown that SG blockade and denervation can reduce the expression of sympathetic neurohormones and the release of noradrenaline prevent the occurrence of cardiac remodeling, fibrosis, and malignant arrhythmia (Gu et al., 2012; Zhang et al., 2019, 2020). However, the side effects of SG blockade and resection limited its clinical application (Goel et al., 2019). A new method of SG denervation after CDs is promising. A variety of proteins in SG plays regulatory roles in cardiac function. After finding out the intervention of the proteins that played vital roles in SG, we can purposefully change the effect of SG on cardiac remodeling after CDs. Therefore, it is important to clarify the proteins involved in the regulation of SG after MI by sequencing. Studies have shown that cardiac nerve sprouting and sympathetic hyperinnervation were more pronounced at 7 days after MI (Zhou et al., 2004). We identified the DEPs in MI groups. Compared with the control group, there were 383 kinds of DEPs totally in the MI group, including 143 upregulated proteins and 240 downregulated proteins. In a further study, the regulating function of the DEPs on cardiac function after MI will be studied.

The GO and KEGG analysis showed that the DEPs were involved in the process of “adrenergic signaling in cardiomyocytes, positive regulation of ERK1 and ERK2 cascade,” and other biological processes. Screening results of the GO and KEGG analysis showed the biological function related to the heart in SGs after MI, including “diabetic cardiomyopathy, fluid shear stress and atherosclerosis, hypertrophic cardiomyopathy, arrhythmogenic right ventricular cardiomyopathy,” and so on. Three of the proteins including TPM1, TPM2, and CALM (meeting the conditions: at least involved in two items, fold change >1.2 or fold change <0.8, and score sequence HT of protein >50) were selected for verification using Western blot experiment.

TPM is a thin filament-associated protein and is associated with morphogenesis, cellular migration, and the regulation of actin filaments (Zhang et al., 2018). TPM1 and TPM2 are two gene subtypes of TPM. TPM1 is an essential sarcomeric component that can stabilize the thin filament and facilitate the interaction of actin with myosin (England et al., 2017). TPM1 plays a vital role in cardiogenesis and is closely related to a variety of CDs including inherited cardiomyopathy and dilated cardiomyopathy (Hershberger et al., 2010; Gupte et al., 2015; Deacon et al., 2019). TPM2 is involved in muscle contraction, cell movement, and other biological processes (Xiong et al., 2018). It is also closely related to CDs (Marshall et al., 2012). Studies have shown that TPM2 exhibited potential as a promising diagnostic and therapeutic biomarker for atherosclerosis (Meng et al., 2019). Moreover, TPM protein also played a role in the nervous system (Gray et al., 2017). CALM is a ubiquitous intracellular Ca^2+^ sensing protein that modifies the gating of numerous ion channels (Chazin and Johnson, 2020). It also has notable roles in cell proliferation, cyclic nucleotide metabolism, cellular Ca^2+^ metabolism, gene expression, muscle contraction, and proteolysis (Sharma and Parameswaran, 2018). CALM activity is closely related to MI, arrhythmia, and hyperthyroid CDs (Beghi et al., 2020; Chazin and Johnson, 2020; Hou et al., 2020) and is involved in the occurrence and development of neurological diseases (O'Day, 2020). Both TPM and CALM proteins played important roles in the cardiovascular system and nervous system at the same time. Proteome sequencing also showed that the expression of TPM1, TPM2, and CALM increased in SGs after MI, and the score sequence HT was the highest among the DEPs related to at least two human CDs.

## Conclusion

We clarified the protein expression in rabbit SG tissue by the TMT quantitative proteomic sequencing and compared the DEPs in the MI group. This study laid a solid foundation for further study on the mechanism of SG in regulating the heart after MI.

## Limitations

This study has some limitations. First, in this study, three proteins related to CDs were selected for validation; however, there may be more important proteins that may play an important role in the development of MI, which should be confirmed in further studies. Second, the SG tissue on the 7th day after MI was used in sequence analysis instead of 1 month after MI when heart remodeling may lead to chronic remodeling of the cardiac nerve sprouting and sympathetic hyperinnervation. Third, the number of samples should be increased in future studies. “Mix the samples” method and the animal-to-animal variation reduced the statistical power of the study.

## Data Availability Statement

The datasets presented in this study can be found in online repositories. The names of the repository/repositories and accession number(s) can be found at: ProteomeXchange, accession no: PXD029386.

## Ethics Statement

The animal study was reviewed and approved by Animal Ethical and Welfare Committee of Chinese Academy Medical Sciences Institute of Radiation Medicine.

## Author Contributions

GL and HF: conception of the work. LC: finishing experiment, analysis data, and drafting of manuscript. HC: submission. XW and TL: data interpretation and critically revised the manuscript. All authors contributed to the article and approved the submitted version.

## Funding

This work was supported by grants from the National Natural Science Foundation of China (No. 82100342), the Tianjin Natural Science Foundation (Nos. 16JCQNJC12000 and 16JCYBJC25000), the China Postdoctoral Science Foundation (No. 2016M601274), the Key Laboratory of Scientific Research Foundation of the Second Hospital of Tianjin Medical University (Nos. 2017ZDSYS07 and 2019ZDSYS14), and clinical study of Second Hospital of Tianjin Medical University (No. 2019LC03).

## Conflict of Interest

The authors declare that the research was conducted in the absence of any commercial or financial relationships that could be construed as a potential conflict of interest.

## Publisher's Note

All claims expressed in this article are solely those of the authors and do not necessarily represent those of their affiliated organizations, or those of the publisher, the editors and the reviewers. Any product that may be evaluated in this article, or claim that may be made by its manufacturer, is not guaranteed or endorsed by the publisher.

## Abbreviations

SG: stellate ganglion
MI: myocardial infarction
TMT: tandem mass tags
KEGG: Kyoto Encyclopedia of Genes and Genomes
GO: gene ontology
TTC, 2, 3: 5-triphenyltetrazolium chloride
LAD: left anterior descending coronary artery
CK: creatine kinase
CK-MB: creatine kinase isoenzyme
LDH: lactate dehydrogenase
PVDF: polyvinylidene fluoride
CALM: calmodulin
TPM1: tropomyosin 1
TPM2: tropomyosin 2
SD: standard deviation
DEPs: differentially expressed proteins
CD: cardiovascular disease.
